# *Streptococcus constellatus* and Prevotella bivia Penile Abscess

**DOI:** 10.1100/tsw.2007.240

**Published:** 2007-10-05

**Authors:** Sandhya Nalmas, Eliahu Bishburg, Trini Chan

**Affiliations:** ^1^ Division of Infectious Disease, Newark Beth Israel Medical Center, Newark, NJ, USA; ^2^ Department of Microbiology, Newark Beth Israel Medical Center, Newark, NJ, USA

**Keywords:** Anginosus, Prevotella, penile abscess

## Abstract

*Streptococcus constellatus* (*S. constellatus*) is known to cause abscesses in the oral, genitourinary, and gastrointestinal tracts, frequently in association with anaerobic bacteria. We report a rare case of *S. constellatus* and *Prevotella bivia* (*P. bivia*) causing a penile abscess, which was successfully treated with surgical drainage and antibiotic treatment.

## BACKGROUND

Genital abscesses requiring hospitalization are common, but penile abscesses are rarely seen in the clinical setting. The cases reported[1] are secondary to urethral catheterization, urethral instrumentation, or originating from pilonidal cysts. Most penile abscesses are caused by mixed aerobic and anaerobic bacteria[2]. Here we report a unique case of penile abscess caused by a rare combination of bacteria *Streptococcus constellatus* and *Prevotella brevia.*

## CASE REPORT

A 44-year-old, African American male was admitted with complaints of marked swelling and pain of his penis for 1 week prior to admission (PTA). The penile lesion started draining yellowish pus 1 day PTA; the patient (pt) received cephalexin and doxycycline. There was no improvement of the swelling or drainage, and the pt developed fever chills and pain on urination. The pt's past medical history is significant for hypertension, obstructive sleep apnea, and mild asthma. The pt claimed to be sexually active with multiple female partners. There was a past history of cocaine use. The pt did not give a history of sexually transmitted disease.

On physical examination, blood pressure was 110/70 mmHg, pulse rate 82/min, and temperature 98.4°F. Genital exam revealed penile swelling, multiple ulcerations with extensive edema and induration of the penile shaft, scrotal swelling extending onto the medial aspect of the right thigh. There was no associated lymphadenopathy. The laboratory examination revealed a white blood cell count of 11,700/mm$^3$ with 77% neutrophils and 13% lymphocytes; hemoglobin 12.3 g/dl, platelet count 388,000/µl; HIV test was negative and RPR test for syphilis was nonreactive. CT of the abdomen and pelvis revealed diffuse inflammatory changes in the scrotum and penis without fluid or air collection. The phallus started draining spontaneously from two sites. The pt underwent incision and drainage of the dorsal aspect of the phallus, which produced necrotic, foul-smelling pus. There was no involvement of the corpora cavernosa. Aerobic and anaerobic cultures were done; anaerobic cultures were taken in port a cul (Becton, Dickinson & Company) and plated immediately. Gram stain of the pus revealed multiple gram-positive cocci and the patient was started on intravenous (i.v.) vancomycin, clindamycin, and cefepime. Culture of the surgical specimen grew *S. constellatus* sensitive to ampicillin, cefepime, cefotaxime, chloramphenicol, clindamycin, ceftriaxone, erythromycin, levofloxacin, and penicillin (Vitek 2 Biomérieux, Hazelwood, MO). The culture was negative for *Neisseria gonorrhea*.

The anaerobic culture grew *P. bivia,* for which sensitivity panel was not placed. Blood cultures remained sterile. Within 24 h after the surgical drainage, there was a marked reduction in the swelling and improvement of the difficulty in urination. The pt was continued on i.v. vancomycin and clindamycin for 2 more days and was discharged on oral amoxicillin/clavulonate 875 mg twice daily for 10 days. The pt was followed up in the urology clinic.

## DISCUSSION

We present the first case of penile abscess caused by *S. constellatus* and *P. bivia,* successfully treated with antibiotics and surgical drainage.

Penile corporeal infection and abscesses have been described in association with cavernous injection and trauma to the penis[3]. One case of spontaneous corporeal abscess was reported in a pt without any associated comorbid conditions[4]. The organisms commonly identified with these abscesses are β-hemolytic streptococci[5] and *Mycobacterium tuberculosis*. Septicemia is relatively rare with penile abscess. Huuskonen and Aaltomaa[6] reported a case of a pt with penile abscess leading to paraphimosis, associated with *Candida albicans* and *Clostridia* in blood cultures. The pt was successfully treated with intravenous fluconazole and ertapenem along with surgical debridement. The most common anaerobes isolated from pt with penile abscess are *Bacteroides fragilis*, *Prevotella* sp., *Porphyromonas* sp., *Clostridium* sp., and *Actinomyces* sp.[7].

The treatment of genital abscesses consists of antibiotics and surgical drainage. The antibiotic treatment of choice for streptococcal abscesses is penicillin G; vancomycin has been used in penicillin-allergic patients. The surgical treatment of the abscesses varies from simple incision and drainage for superficial abscesses, to extensive debridement and CT-guided drainage if the abscess is deep seated. Anaerobes are commonly treated with metronidazole, amoxicillin/clavulanate, ampicillin/sulbactam, piperacillin/tazobactam, imipenem, and clindamycin[8].

*S. constellatus* is part of the *Streptococcus milleri* (*S. milleri*) group. The *S. milleri* group consists of *S. anginosus*, *S. constellatus,* and *S. intermedius*[9], which is now included in the anginosus group. These organisms are part of the natural flora found in the oral, gastrointestinal, and genitourinary tracts[10]. The organisms appear on gram stain as gram-positive cocci and can be α-, β-, or nonhemolytic on blood agar. Members of this group belong to Lancefield groups A, C, F, or G[11]. The production of hyaluronidase by these bacteria contributes to the abscess formation[13]. These organisms are usually sensitive to β-lactam antibiotics, such as penicillin, ceftriaxone and ampicillin, doxycycline, erythromycin, vancomycin, and the aminoglycosides, and intermediately sensitive to clindamycin. Increased resistance to penicillin and clindamycin has been described[12,14,15]. *S. constellatus* causes superficial abscesses often isolated with anaerobic bacteria[16]. These superficial abscesses are generally associated with injection of illegal drugs[16]. The most common site of involvement is skin and soft tissue, followed by intra-abdominal and perirectal regions. Genitourinary abscesses with *S. constellatus* are rare, reported in one study, and isolated from 2 of 41 abscesses[16].

*Prevotella* sp. is a gram-negative anaerobic rod and is related to *Bacteroides* sp. It differs from *Bacteroides* in its inability to grow in the presence of bile and to hydrolyze esculin. *Prevotella* sp. produces β-lactamase and is usually susceptible to clindamycin, metronidazole, imipenem, and amoxicillin/clavulonate[18]. Increased resistance of *Prevotella* to clindamycin has been noticed globally[17].

*Prevotella* sp. is part of cervical flora, and causes perirectal abscesses[19] and OBG infections, including PID infection[10], in the presence of aerobes thought to increase its pathogenesis[16,20].
